# Anticoagulation in Frail Older Adults with Non-Valvular Atrial Fibrillation: Clinical Challenges and Personalized Approach

**DOI:** 10.3390/jcm14228079

**Published:** 2025-11-14

**Authors:** Elisa Fabbri, Lorenzo Maestri, Paolo Muratori

**Affiliations:** 1 Department of Medical and Surgical Sciences, University of Bologna, 40138 Bologna, Italy; 2 Division of Internal Medicine, Morgagni-Pierantoni Hospital, 47121 Forlì, Italy

**Keywords:** atrial fibrillation, anticoagulation, older adults, frailty, frail, DOACs, comprehensive geriatric assessment, bleeding risk

## Abstract

The prevalence of atrial fibrillation (AF) is increasing and often coexists with frailty. The management of anticoagulation therapy in frail older adults with AF is especially challenging due to the high risk of bleeding complications. The aim of this narrative review is to provide a comprehensive overview of current evidence about the management of anticoagulation in frail older adults with non-valvular AF. First, frailty itself should not be considered a contraindication. A comprehensive geriatric assessment is recommended to identify and potentially address conditions that may increase the risk of bleeding, such as inappropriately prescribed medications or malnutrition. Overall, the net clinical benefit remains in favour of oral anticoagulation in frail older adults, even if it decreases with increasing frailty severity. Direct Oral Anticoagulants (DOACs) show a better effectiveness and safety profile compared with Vitamin K antagonists (VKAs) in this population. Among DOACs, apixaban seems to be the safest. Also, edoxaban at a very low dosage (15 mg/day) could be an effective therapy in patients for whom the standard anticoagulation is contraindicated. Moreover, switching from VKAs to DOACs in frail older adults is a complex decision and should be personalized according to the stability of the ongoing anticoagulant therapy, the bleeding risk profile, and the severity of frailty. Finally, although further studies are required to confirm their effectiveness, factor XIa inhibitors are emerging as new promising alternative therapies because they have been associated with a lower bleeding risk compared with DOACs.

## 1. Introduction

The global population is aging, and this trend will continue over the coming decades. As a result, the prevalence of atrial fibrillation (AF) is increasing and expected to reach nearly 18 million cases in Europe by 2060 [1]. In fact, advanced age is a major risk factor for AF, which affects approximately one in three individuals aged 85 years or older [2] and can be considered in all respects a geriatric syndrome [3].

Aging also plays a crucial role in the development of frailty, a multisystem geriatric condition defined by diminished physiological reserves and increased vulnerability to adverse outcomes [4,5]. Therefore, AF and frailty frequently occur simultaneously [6], with nearly 40% of patients with AF also experiencing frailty, as revealed by a recent meta-analysis [7], and show a bidirectional relationship. Specifically, the biological aging process that contributes to frailty also drives the cardiac structural and functional alterations that lead to the onset of AF. Furthermore, frailty raises the likelihood of hospitalization due to AF [8] and increases the risk of adverse outcomes [7,9,10]. Conversely, AF increases the risk of frailty and accelerated functional decline among older individuals [11].

Notably, cardioembolic stroke is one of the most feared complications of AF, and anticoagulation therapy is essential to prevent it. In frail older adults, treatment decisions are more challenging due to a higher risk of bleeding complications. These patients often have multiple co-morbidities and take numerous medications, like aspirin or anti-inflammatory drugs, that further elevate the bleeding risk. Moreover, evidence is limited in frail older adults due to their frequent exclusion from randomized clinical trials (RCTs). Thus, frail older adults with complex clinical profiles, even in the absence of a clear contraindication, are less likely to receive [12,13,14] or continue [15] anticoagulation therapy. The introduction of Direct Oral Anticoagulants (DOACs) has not completely resolved the treatment gap between frail and non-frail patients with AF [14,16]. Almost one in four older adults treated with DOACs is inappropriately underdosed [17]. Also, discontinuation of oral anticoagulation without a clear contraindication seems to be associated with a higher risk of mortality in clinically complex older adults [18].

Therefore, optimizing care for frail older adults with AF requires a personalized strategy that incorporates multidimensional geriatric assessment and a thorough evaluation of risks and benefits. Due to the enormous relevance of this topic for the scientific community, literature examining the clinical benefits of anticoagulation in frail older adults and comparing different anticoagulation agents in this special population is continuously growing. The purpose of this review is to provide a comprehensive overview of current evidence about the management of anticoagulation in frail older adults with non-valvular atrial fibrillation up to October 2025.

## 2. Methods

This narrative review summarizes current evidence regarding anticoagulation therapy in frail older adults with non-valvular AF. Specifically, a comprehensive literature search on this topic was conducted on PubMed (Bethesda, MD, USA). Keywords included: “atrial fibrillation”, “frailty”, “older adults”, “multimorbidity”, “polyfarmacy”, “anticoagulation”, “VKAs”, “warfarin”, “DOACs”, “Apixaban”, “Edoxaban”, “Rivaroxaban”, “Dabigatran”, “low dose”, and “switching”. The evaluation incorporates randomized clinical trials (RCTs), observational studies, meta-analyses, systematic reviews, and clinical guidelines published between January 2010 and October 2025, with earlier articles highlighting enduring concepts as supplements. The literature published in the last decade was prioritized. More than 400 papers were screened. Non-English articles were not considered. The authors examined the titles and abstracts of the collected papers and their references to identify qualifying articles. Full texts of all pertinent papers were obtained. In the end, 131 references were determined to serve as supporting evidence.

Moreover, RCTs, observational studies, and meta-analyses investigating the net clinical benefit of oral anticoagulation in frail older adults, as well as comparing different anticoagulant agents (DOACs versus warfarin, or DOAC versus DOAC) in this special population, were selected and are summarized in Tables. Noteworthy, studies that did not directly assess frailty were excluded from this selection.

## 3. Frailty: Definition and Assessment

Frailty is a geriatric syndrome characterized by diminished resiliency and reduced physiological reserves that increase susceptibility to stressors and elevate the risk of adverse outcomes and mortality [4,5]. Research conducted in recent decades indicates that the mechanisms of accelerated aging at cellular and subcellular levels, such as chronic inflammation, mitochondrial dysfunction, epigenetic alterations, and cellular senescence, the so-called “hallmarks of aging”, cause progressive damage accumulation and loss of resilience [5]. These are responsible for homeostatic dysregulation and reduced physiological reserves and function across multiple organs and systems, ultimately leading to the clinical manifestation of frailty and its association with age-related cardiovascular diseases [19], such as AF [20].

Over the past decades, many different tools to assess frailty have been developed. However, two main conceptualizations of frailty prevail in the literature [5]. Fried defined the physical phenotype of frailty based on a vicious circle of declining energy and reserves, marked by loss of muscle mass and strength, reduced resting metabolic rate, energy expenditure, and mobility, leading to progressive decline in health and function [21]. Conversely, Rockwood elaborated a different approach, measuring frailty as the accumulation of deficits [22]. The original Frailty Index (FI) included 70 items, from symptoms and signs to laboratory anomalies, diseases, geriatric syndromes, and disabilities [23] and the ratio of deficits present to the total number considered indicates the likelihood of frailty. Multiple shorter versions of FI have since been developed. Despite their differences, both Fried’s and Rockwood’s measures strongly predict adverse outcomes in older adults [21,23,24]. Additionally, the Clinical Frailty Scale (CFS) is a quick and easy test, based on clinical judgement, that could be used to predict adverse outcomes in hospitalized older patients [25].

## 4. Anticoagulation in Frail Older Adults with Atrial Fibrillation

Older adults (≥65 years old) account for about 80% of individuals with AF, and the prevalence of AF significantly increases with advanced age [26]. Older adults are more likely to experience frailty, multimorbidity, and polypharmacy [27].

Both thrombotic and hemorrhagic risks increase with age, but the risk of stroke increases with age more than the risk of bleeding [28]. International guidelines recommend that the decision to prescribe oral anticoagulation therapy should be guided by the patient’s overall thromboembolic risk, not by age alone [29,30]. Validated risk assessment tools should be used by clinicians to support their decisions. For example, the CHA_2_DS_2_-VA score (Supplemental Table S1), an evolution of the CHA_2_DS_2_-VASc score that excludes gender, is recommended by the 2024 European Society of Cardiology (ESC) guidelines to assess thromboembolic risk. Specifically, a score of 2 or more indicates high thromboembolic risk and supports anticoagulation therapy, while a score of 1 suggests that oral anticoagulation may be considered. As the thromboembolic risk increases with advancing age, the CHA_2_DS_2_-VA score assigns 1 point for ages 65 to 74 and 2 points for age 75 or older. Thus, age 75 or above independently qualifies as a high-risk factor. Although the risk of bleeding increases with age, guidelines advocate that advanced age alone should not justify withholding or discontinuing anticoagulation. Indeed, a thorough assessment of bleeding risk to identify and possibly correct modifiable factors is recommended. Consistently, in the ACONVENIENCE Study, a multidisciplinary panel of experts agreed that advanced age should not influence the decision regarding anticoagulation. Instead, the decision should be based on a careful evaluation of net clinical benefit during the comprehensive geriatric assessment [31].

The presence of frailty is associated with a higher rate of adverse outcomes in older adults with AF. In particular, both high CHA_2_DS_2_-VASc and high HAS-BLED scores are strongly associated with frailty [32]. However, according to current guidelines, frailty itself should not be considered a contraindication to anticoagulation therapy [30,33]. It is rather recommended a personalized approach, based on multidimensional evaluation, an accurate balance of risks and benefits, and an integrated and patient-centered management to optimize the treatment of comorbidities and reduce the risk of bleeding. Nevertheless, concerns regarding hemorrhagic complications in frail older adults are common among clinicians. Consequently, even in the absence of clear contraindications, frail older patients with AF are frequently undertreated, receive inappropriately reduced dosages, or have anticoagulant therapy discontinued [14,34]. Cognitive impairment, loneliness, and poor compliance commonly represent barriers to prescription. Also, the co-existence of multiple chronic conditions with polypharmacy and the consequent increased risk of drug-drug interactions often discourages clinicians from prescribing anticoagulation. Previous falls or a high risk of falls may represent an additional concern. Furthermore, decision-making is challenging in this population due to the paucity of studies about the clinical benefit of anticoagulation in frailty. Indeed, frail older adults are substantially underrepresented in RCTs. Reasons include concerns about the bleeding risk, patient complexity, difficulties in recruitment and adherence, and the lack of a standardized definition of frailty. The consequences are the shortage of specific data and the weakness of evidence in this population. However, a few observational and real-world studies have investigated the benefit of anticoagulation in very elderly or frail older adults [28,35,36,37,38]. In particular, Shah and colleagues pointed out that the net clinical benefit of oral anticoagulation decreases in very old people [36]. Only two studies have examined the net clinical benefit of oral anticoagulation specifically in frail older adults (Table 1). Specifically, a Korean retrospective study, enrolling more than 80 thousand frail participants, found that oral anticoagulation was associated with lower risk of ischemic stroke and cardiovascular death, with no significant difference in the risk of major bleeding [37]. Moreover, a Danish cohort study showed that the net clinical benefit of anticoagulation remains in favour of anticoagulation in frail older adults, even if it decreases for advancing age and increasing frailty [38]. Both studies evaluated frailty using the Hospital Frailty Risk Score, which was validated for individuals ≥ 75 years to pinpoint those at a greater risk of mortality or re-admission within 30 days post-discharge. Nevertheless, the variety of available assessment tools makes it unclear which instrument is most effective for evaluating frailty. Furthermore, observational studies face important limitations since the reasons behind the decision not to prescribe or to stop oral anticoagulation may not always be identified, and findings could be skewed by the fact that older adults receiving oral anticoagulation therapy often tend to be healthier and more robust compared to those who are not prescribed the treatment.

In this regard, a few observational studies found that discontinuation of oral anticoagulant therapy was associated with a higher risk of mortality and adverse outcomes in frail older adults, highlighting the complexity of anticoagulation decision-making in this special population [18]. However, other researchers found that discontinuing oral anticoagulation in very frail older patients during hospitalization was linked to an increased overall mortality rate, without a significant rise in the risk of stroke or a significant decline in bleeding risk [39]. In a non-randomized setting, the decision to stop oral anticoagulation is likely influenced by the anticipated poor net clinical benefit due to a comprehensive geriatric assessment that predicts a short life expectancy. This might largely account for the increased mortality risk observed.

**Table 1:** Studies investigating the net clinical benefit of oral anticoagulation (OAC) in frail older adults with atrial fibrillation (AF).

First Author, Year	Country	Study Population	Mean Age	Frailty Definition	Main Results	Conclusion
Kim, 2022 [37]	Korea	83,635 frail patients with AF - 34.1% OAC - 65.9% no OAC	78.5 years old	≥5 Hospital Frailty Risk Score	OAC use vs. non-use Outcome HR (95% CI): NACE 0.70 (0.66–0.74) Ischemic Stroke 0.91 (0.85–0.97) Major bleeding 1.05 (0.99–1.11) CV death 0.40 (0.36–0.44)	OAC treatment was associated with a positive net clinical outcome.
Søgaard, 2024 [38]	Denmark	36,223 frail patients with AF - 61.8% OAC - 38.2% no OAC	79 years old	≥5 Hospital Frailty Risk Score	NCB 0.70% (95% CI, 0.32–1.08%)	The NCB was in favor of anticoagulation, but decreased with advancing age and increasing frailty.

NACE = net clinical adverse event; NCB = net clinical benefit.

## 5. Bleeding Risk and Integrated Approach in Older Adults with AF

**Figure 1:**
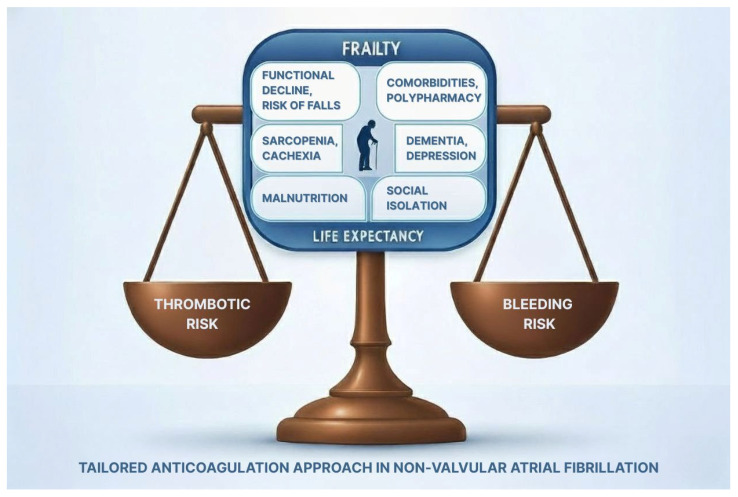
Integrated and tailored approach to anticoagulation prescription in older patients with atrial fibrillation, based on the comprehensive geriatric assessment and life-expectancy evaluation.

Frailty is associated with increased risk of stroke in older patients with AF, but at the same time, it significantly increases the risk of bleeding, which is why frail older adults are often undertreated. However, as previously highlighted in this review, frailty itself is not a sufficient criterion for withholding or discontinuing anticoagulation therapy in older adults. A personalized and integrated clinical approach by a multidisciplinary team, based on a comprehensive geriatric assessment (CGA) and tailored to the patient (Figure 1), is required to identify and eventually address conditions that may increase the risk of bleeding [40].

In recent decades, the prevalence of multiple chronic conditions has risen substantially in individuals with AF [41]. Notably, the risk of adverse outcomes and mortality significantly rises for an increasing number of diseases [42,43], while, simultaneously, the probability of receiving an anticoagulant prescription linearly declines in relation to the number of concurrent chronic conditions [44]. Both the number and the type of comorbid diseases may influence the decision-making and the selection of the anticoagulation treatment. One of the most challenging clinical scenarios involves older patients who have both AF and cancer [45]. Older patients with cancer are often frail and present an increased risk of bleeding and higher mortality. Metastatic disease, gastrointestinal cancer, and the presence of thrombocytopenia are established bleeding risk factors. Moreover, in older patients with or without cancer, the co-occurrence of severe chronic kidney disease is associated with a worse prognosis [46], with an increase of both thrombotic and hemorrhagic risk. Furthermore, anaemia is a common condition in older adults and typically arises from multiple factors. Notably, anaemia often co-occurs with AF in older adults and is correlated with an increased risk of negative outcomes and bleeding, necessitating close monitoring during anticoagulation treatment [47].

Polypharmacy is closely linked to multimorbidity. The primary concern is the heightened risk of drug-drug and drug-disease interactions, which significantly increases the likelihood of adverse events. A recent meta-analysis reported a 53.5% prevalence of polypharmacy in older patients with AF, with significant associations to major bleeding, intracranial bleeding, and mortality [48]. A Swedish cohort study further found that both inappropriate prescriptions and prescribing omissions raised the risk of adverse outcomes, including cardiovascular and overall mortality, hospitalizations, stroke, bleeding, and falls in older adults with AF and multimorbidity [49]. Benzodiazepines were the most common inappropriate prescriptions, while anticoagulants were the most frequently omitted [49]. Therefore, a careful pharmacological revision and reconciliation process is required in older adults with AF.

Moreover, it is well-known that individuals with AF have an increased risk of cognitive decline and dementia [50]. While there is some evidence indicating that oral anticoagulants, especially DOACs, could help prevent cognitive impairment [51,52,53,54], the issue is still debated. Indeed, recent results from the randomized BRAIN-AF trial, which were presented at the American Heart Association Scientific Sessions in Chicago in November 2024 and have not yet been published, showed that rivaroxaban did not succeed in preventing cognitive decline when compared to placebo in patients with AF [55]. Cognitive decline and dementia frequently come with significant challenges and doubtful advantages when it comes to anticoagulant therapy, as they are linked to an increased risk of bleeding and higher mortality rates. Particularly, anticoagulation seems to provide a net mortality advantage for patients with AF and early-stage dementia, but its application in very elderly individuals with advanced dementia needs careful, personalized evaluation of patient objectives and the risk/benefit balance [56]. Furthermore, depression, a common condition in older adults with AF, increases the risk of cognitive impairment in these patients [57], and it could lead to decreased adherence to anticoagulation therapy [58].

Social isolation and loneliness are also associated with lower medication adherence and linked to a worse prognosis in older patients with AF [59]. Therefore, the evaluation of the socio-familial status is an essential part of comprehensive geriatric assessment before decision-making about the prescription of anticoagulant therapy.

Additionally, one of the most pervasive features of aging is the loss of physical function, determined by the progressive decline of muscle mass (sarcopenia) and muscle strength at the advanced stage of life. Loss of strength and reduced physical performance, specifically slowness of gait, are key components of the physical phenotype of frailty [21]. A recent study found that AF negatively impacts physical performance in older adults [60]. Furthermore, a history of falls is associated with a higher risk of death, cardiovascular events, thromboembolism, and major bleeding among older adults with AF [61].

Besides, malnutrition is a risk factor for AF development, and it is associated with cachexia and poor prognosis in older adults with AF [62]. In these patients, malnutrition increases both the thrombotic and the bleeding risk, making anticoagulant treatment decisions especially difficult [63].

The importance of a holistic approach and integrated care for managing AF patients is well-documented in the literature and endorsed by various international guidelines [29,30]. The ABC pathway, an integrated approach focusing on stroke prevention, symptom management, and comorbidities prevention and treatment [64], has been shown to significantly lower the risk of negative outcomes in older adults with AF [65,66]. Recently, the European guidelines updated this strategy by introducing the CARE-AF pathway, which further prioritizes the holistic management of comorbidities, placing it in first position [30]. Moreover, several ongoing European projects are focused on the design and implementation of new integrated care pathways for AF patients with multiple co-morbidities [67,68]. Of relevance, the AFFIRMO Programme is a current European initiative aimed at assessing the efficacy of patient-centered and integrated care for older patients with AF and multiple health conditions, by incorporating CGA into the ABC pathway to offer a more thorough evaluation of the patient’s health and a more accurate identification of the presence and severity of frailty [67]. Specifically, the CGA, with a comprehensive evaluation of multiple domains that define health and well-being in older adults (physical health with special regard to comorbidities, cognitive health, physical function, nutritional status, and social environment), may help to better identify and eventually address bleeding risk factors in frail older adults with AF and support decision-making about anticoagulation.

It is important to highlight that thrombotic and bleeding risks evolve with time and aging. Thus, it is essential to assess bleeding risk in older adults with AF using CGA before starting anticoagulation therapy, and to conduct regular follow-ups to monitor and address new or increasing risks as they age.

Finally, evaluating the remaining life expectancy is crucial before deciding on anticoagulation for older adults with AF. Parks and colleagues recommend that stopping treatment should be considered for patients nearing the end of life (defined by a life expectancy of less than one year due to terminal conditions) since the potential harm may surpass the advantages [26]. Naturally, the decision-making process should involve both the patient and their family, considering their preferences.

## 6. Selection of the Anticoagulant Agent

### 6.1. Vitamin K Antagonists (VKAs)

Vitamin K antagonists (VKAs), particularly warfarin, have been for a long time the principal drugs to prevent thromboembolism in patients with AF. VKAs inhibit hepatic synthesis of coagulation factors II, VII, IX, and X. Due to their slow onset of action, bridging with low-molecular-weight heparin is required. The narrow therapeutic index of VKAs necessitates regular monitoring of coagulation parameters, such as the International Normalized Ratio (INR). Specifically, VKAs are effective only if time in therapeutic range (TTR) is maintained >70%. Notably, sensitivity to VKAs increases with age [69], leading to a greater risk of bleeding and the need for closer monitoring. Furthermore, these drugs have numerous dietary and pharmacological interferences that need to be considered, especially in patients with multimorbidity and polypharmacy, where the risk of drug-drug interactions is elevated. In case of major bleeding while on warfarin, the reversal agent is vitamin K, which acts gradually (over several hours). Therefore, the use of prothrombin complex concentrate (PCC) is recommended [30].

### 6.2. Direct Oral Anticoagulants (DOACs)

Over the past 15 years, DOACs have been implemented as an alternative to VKAs for anticoagulation in patients with AF. Specifically, dabigatran is a direct inhibitor of thrombin, while rivaroxaban, apixaban and edoxaban inhibit Factor Xa. Large RCTs demonstrated at least non-inferior efficacy of DOACs compared to warfarin in relation to the primary end point (prevention of thromboembolic events and ischemic stroke), with a more favourable safety profile (specifically, a greater reduction of intracranial haemorrhages) [70,71,72,73]. Therefore, current international guidelines on atrial fibrillation anticoagulation therapy recommend preferring DOACs to VKAs, except in patients with mechanical heart valves or moderate-to-severe mitral stenosis [30].

RCTs on DOACs in AF included a relevant proportion (around 30–40%) of patients aged 75 years or older [74]. Subgroup analyses showed that all DOACs consistently maintain at least non-inferiority compared to warfarin across different age groups [75,76,77,78]. Moreover, apixaban and edoxaban showed a significantly better safety profile in patients aged 75 or older compared to warfarin [77,78]. Several meta-analyses confirm that DOACs are associated with better outcomes than warfarin in older adults, including those aged 80 years or older [79,80,81,82,83,84,85,86,87]. Consistent results were also observed in real-world studies [88,89,90].

Furthermore, among DOACs, apixaban demonstrated the lowest risk of bleeding [91]. Noteworthy, a recent, real-world study found that edoxaban had a similar effectiveness but is associated with a higher risk of bleeding compared to apixaban in very elderly patients (≥80 years) [92].

These findings align with the 2023 American Geriatrics Society (AGS) Beers Criteria [93], which recommend using DOACs as initial therapy for older patients with atrial fibrillation rather than warfarin. Notably, according to the 2023 AGS Beers Criteria, in older patients, apixaban should be preferred among the DOACs due to its superior safety profile, while rivaroxaban should be avoided due to its increased risk of major and gastrointestinal bleeding [93]. However, further studies comparing DOAC-DOAC in older adults are required to confirm and strengthen these recommendations.

Interestingly, in older patients with AF and dementia, DOACs seem to offer a safer profile regarding major bleeding compared to Vitamin K antagonists (VKAs) [94].

However, RCTs regarding the effectiveness and safety of DOACs specifically in frail patients are limited. Two post-hoc subgroup analysis of the ARISTOTELE trial, using multimorbidity [95] and polypharmacy [96] respectively as proxies of frailty, demonstrated that apixaban exhibited a superior safety profile compared to warfarin, even among patients with the greatest burden of multiple chronic conditions [95]. However, the benefit of apixaban over warfarin on major bleeding diminished as the number of concomitant medications increased [96]. Additionally, a subgroup analysis of the ENGAGE AF-TIMI 48 trial indicated that edoxaban was associated with a lower risk of severe bleeding and all-cause mortality compared to warfarin in patients at risk of falling [97].

Notably, a further post-hoc analysis demonstrated that edoxaban was safer than warfarin across various levels of frailty, except for severe frailty [98].

This analysis and other findings from observational studies [99,100,101,102], including specifically frail older adults, are presented in Table 2. In particular, a cohort study analyzing Medicare data found that apixaban was associated with lower rates of adverse events across all frailty levels compared to warfarin [100]. Moreover, a large cohort study found that both standard and reduced DOAC regimens were associated with similar thromboembolism risk and significantly lower bleeding risk compared to warfarin in frail patients with AF [103]. Finally, a meta-analysis confirmed that DOACs showed better effectiveness and safety than warfarin in AF patients with frailty [104].

Evidence regarding DOAC-DOAC comparison in frail older adults with AF is very limited. Specifically, a sub-analysis of the ARISTOPHANES study enrolling frail older adults demonstrated that apixaban was associated with significantly reduced risk of major bleeding compared to warfarin, dabigatran, and rivaroxaban [101]. However, further studies are required to provide more robust recommendations.

Finally, in case of major bleeding while on oral anticoagulation, DOACs have specific antidotes (idarucizumab for dabigatran; andexanet alfa for factor Xa inhibitors), which work rapidly (within minutes). Guidelines recommend using PCC when those antidotes are unavailable [30]. A recent RCT comparing andexanet alfa to usual care in patients experiencing acute intracranial hemorrhage (average age 78.9 years) demonstrated that andexanet alfa resulted in a better control of hematoma expansion [105]. To the best of our knowledge, there are no RCTs comparing DOACs with their antidotes against warfarin with PCC regarding mortality and rapid haemostasis outcomes in older adults.

**Table 2:** Studies comparing the efficacy and safety of different oral anticoagulation agents in frail older adults with atrial fibrillation (AF).

First Author, Year	Study Design	Study Population	Frailty Definition	Results
Martinez, 2018 [99]	Retrospective study	Three cohorts of frail patients: - Apixaban vs. warfarin (n = 1392 per group) - Dabigatran vs. warfarin (n = 1350 per group) - Rivaroxaban vs. warfarin (n = 2635 per group)	Johns Hopkins Claims-based Frailty Indicator score ≥0.20	Rivaroxaban, but not apixaban or dabigatran, was associated with a significant hazard reduction of stroke or systemic embolism compared with warfarin. No significant differences in major bleeding were found between DOAS and warfarin.
Wilkinson, 2020 [98]	Post hoc analysis of the ENGAGE TIMI 48 RCT	20,867 patients randomized at edoxaban vs. warfarin	Frailty index - 0 to <0.12 fit - ≥0.12 to <0.24 pre-frail - ≥0.24 to <0.36 mild-moderate - ≥0.36 to 1.0 severe	Edoxaban was associated with similar efficacy to warfarin in every frailty category and was associated with lower rates of bleeding except in those with severe frailty.
Kim, 2021 [100]	Retrospective cohort study	3 cohorts of dabigatran (n = 81,863) vs. warfarin (n = 256,722); rivaroxaban (n = 185,011) vs. warfarin (n = 228,028); apixaban (n = 222,478) vs. warfarin (n = 206,031)	Claims-based frailty index - <0.15 non-frail - ≥0.15 to <0.24 pre-frail - ≥0.25 frail	Apixaban was associated with lower rates of the composite endpoint of death, ischemic stroke, or major bleeding across all frailty levels. Dabigatran and rivaroxaban were associated with lower event rates only among nonfrail patients. This beneficial association for apixaban vs. warfarin in the frail subgroup appeared to be mainly driven by a large reduction in major bleeding.
Lip, 2021 [101]	Subgroup analysis of the ARISTOPHANES study (retrospective study)	150,847 patients, grouped into six cohorts: - apixaban-warfarin, - apixaban-rivaroxaban, - apixaban-dabigatran, - dabigatran-warfarin, - dabigatran-rivaroxaban, - rivaroxaban-warfarin	Claims-based frailty index ≥ 0.20	DOAC–warfarin comparison Apixaban and rivaroxaban were associated with a lower risk of stroke/systemic embolism compared with warfarin. Regarding major bleeding, apixaban and dabigatran were associated with a lower risk, while rivaroxaban was associated with a higher risk compared with warfarin. DOAC–DOAC comparison Regarding stroke and systemic embolisms, apixaban presented a similar risk compared with dabigatran and a lower risk compared with rivaroxaban, while dabigatran was associated with a similar risk compared with rivaroxaban. Regarding major and gastrointestinal bleeding, apixaban was associated with a lower risk compared with dabigatran and rivaroxaban, while dabigatran was associated with a lower risk compared with rivaroxaban. Overall, apixaban was the OAC with the best safety profile.
Zheng, 2022 [104]	Meta-analysis of four studies [91,92,93,94]	A total of 835,520 patients		Compared with warfarin, DOACs were significantly associated with reduced risks of - stroke or systemic embolisms (HR = 0.79, 95% CI: 0.69–0.90), - ischemic stroke (HR = 0.79, 95% CI: 0.71–0.87), - hemorrhagic stroke (HR = 0.52, 95% CI: 0.35–0.76), - all-cause death (HR = 0.90, 95% CI: 0.84–0.96) - major bleeding (HR = 0.79, 95% CI: 0.64–0.97) - intracranial hemorrhage (HR = 0.58, 95% CI: 0.52–0.65) - No statistical differences were found in gastrointestinal bleeding between DOACs and warfarin.
Lin, 2023 [102]	Retrospective cohort study	45,950 apixaban, 45,320 rivaroxaban, and 45,281 warfarin initiators	claims-based frailty index - <0.15 non frail - ≥0.15 and <0.25 pre-frail - ≥0.25 frail	Apixaban was associated with lower rates of the composite endpoint of ischemic stroke, systemic embolism, major bleeding, or death than rivaroxaban and warfarin, especially for those with frailty
Søgaard, 2024 [103]	Retrospective cohort study	32,048 frail patients, divided into three groups - starting warfarin - starting standard dose DOACs - starting reduced dose DOACs	Hospital Frailty Risk Score ≥ 5	A similar thromboembolic risk was found between DOACs (either standard or reduced dose) and warfarin. Major bleeding was significantly lower with both standard and reduced DOAC doses compared with warfarin.

HR = Hazard ratio.

## 7. Appropriate Dose

Unlike VKAs, which require dose adjustments based on INR monitoring, DOACs are administered at fixed doses. Dose-reduction criteria (Supplementary Materials Figure S1), as defined in their respective registration trials [70,71,72,73], necessitate strict adherence to maintain the balance between efficacy and safety. However, pharmacokinetic and pharmacodynamic changes associated with aging and frailty add significant complexity, requiring a clinical approach that goes beyond the straightforward application of dosing algorithms [74].

A well-established limitation is the use of the Cockcroft-Gault formula to estimate creatinine clearance (CrCl) in sarcopenic individuals [106]. The reduced muscle mass leads to lower creatinine production, which can cause a significant and potentially dangerous overestimation of renal function. Although Cystatin C-based equations for estimating Glomerular Filtration Rate (eGFR) are considered more accurate in this population, the 2024 ESC guidelines have not yet incorporated them as a standard for DOAC dose adjustment. Nonetheless, its measurement is strongly encouraged in clinical practice for equivocal cases, particularly when a patient exhibits features of frailty and sarcopenia but has a calculated CrCl near the upper threshold for dose reduction.

Emerging evidence indicates that frailty and its associated sarcopenia can significantly alter the pharmacokinetics of DOACs, as hydrophilic drugs [107]. Specifically, a marked reduction in muscle mass decreases the drug’s volume of distribution. Consequently, a standard fixed dose may result in higher plasma concentrations and an excessive anticoagulant effect. This was confirmed in a prospective study where lower appendicular lean mass was independently associated with supratherapeutic anti-Xa levels, even after adjusting for age, total weight, and renal function [108].

Further research corroborates this concern. One study found that dose-normalized through plasma concentrations of apixaban were 2.48 times higher in frail participants (defined by Fried’s criteria) compared to their robust counterparts. Notably, drug concentrations were also significantly elevated in patients who exhibited only weak grip strength or slow gait speed—two core components of the frailty phenotype. These findings suggest that direct measures of physical performance may be more sensitive predictors of DOAC accumulation risk than the standard dose-reduction criteria alone [107].

Inappropriate DOAC dosing in frail older patients with AF remains prevalent, particularly off-label underdosing, which occurs in 17% to 30% of cases [109,110,111,112]. The consequence of the inappropriate DOACs underdosing is an increased incidence of thromboembolic events [113], without a significant decrease in major bleeding events [114,115]. Underdosing of apixaban, in particular, has been independently linked to an elevated risk of mortality [116]. Consistently, the standard dose of apixaban resulted in a safe, effective, and suitable treatment for patients presenting with a single dose-reduction criterion, such as advanced age, low body weight, or renal dysfunction [117]. These findings highlight that reducing DOAC doses without a clear, evidence-based indication is an ineffective strategy for improving patient safety and significantly compromises therapeutic efficacy. A specific therapeutic challenge arises in managing very elderly patients with atrial fibrillation who are ineligible for standard anticoagulation due to an exceptionally high bleeding risk. For this select population, the ELDERCARE-AF trial provided crucial evidence demonstrating that edoxaban 15 mg once daily against placebo in patients aged ≥80 years who were not candidates for standard therapy was effective in preventing stroke or systemic embolism, without a significant increase in major bleeding [118]. Subsequent analyses confirmed that these benefits were consistent across various high-risk subgroups, including patients with significant frailty [119], low body weight [120], or moderate renal impairment [121]. These findings establish an evidence-based therapeutic niche for edoxaban 15 mg as a viable stroke prevention strategy in this specific, vulnerable population for whom standard anticoagulation is contraindicated [118].

## 8. Switching from VKAs to DOACs

Transitioning from VKAs to DOACs represents a current major issue in the management of AF, particularly in older and frail populations, where the balance between thromboembolic prevention and bleeding risk is especially delicate. In the previous paragraphs, we already pointed out that current international guidelines favor DOACs over VKAs for stroke prevention in non-valvular AF. Therefore, switching to DOACs could be considered for patients already treated with VKAs. In this regard, guidelines recommend switching to a DOAC in patients unable to maintain an adequate time in therapeutic range (TTR < 70%) or when concerns regarding intracranial hemorrhage arise. However, a specific recommendation (Class IIb) suggests that continuing VKA therapy may be considered in clinically stable patients aged ≥75 years with significant polypharmacy, in order to minimize bleeding risk [30]. This recommendation is largely based on the results of the FRAIL-AF trial, the only large-scale randomized controlled trial to specifically evaluate switching from VKAs to DOACs in frail older adults [122]. The study enrolled patients aged ≥75 years with frailty (Groningen Frailty Indicator score ≥ 3) who were on stable and therapeutic VKA treatment. In this trial, switching to a DOAC was associated with a significantly higher composite primary bleeding endpoint compared with VKA continuation, without a significant reduction in thromboembolic events, leading to the early termination of the trial for futility [122]. These findings suggest that in frail older patients who are stable on well-managed VKA therapy, switching to a DOAC may increase bleeding risk without providing a short-term thromboembolic benefit. Notably, rivaroxaban comprised 50.2% of the DOAC prescriptions in this trial, an agent that some observational studies have associated with a higher risk of bleeding, particularly gastrointestinal bleeding, compared to other DOACs [123,124]. These data must be contextualized within the broader evidence base. A post-hoc sub-analysis of the COMBINE-AF database, which pooled data from over 71,000 patients from the four pivotal DOAC registration trials, offered a different perspective [125]. In older (≥75 years), frail, VKA-experienced patients, compared with warfarin, standard-dose DOACs demonstrated reductions in stroke or systemic embolism and all-cause mortality that were similar to those observed in non-frail patients. Moreover, in this population, major bleeding was similar for standard dose DOACS versus warfarin. Specifically, DOACs significantly reduced intracranial and fatal bleeding, although the risk of gastrointestinal bleeding was increased in older, frail VKA-experienced patients [125]. The authors concluded that standard-dose DOACs remain a reasonable choice in this population, albeit requiring careful attention to the patient’s bleeding phenotype [125]. In conclusion, while DOACs are generally preferred over VKAs for AF, the decision to switch from stable VKA therapy to a DOAC in frail, elderly individuals remains complex. The therapeutic choice necessitates a careful assessment of patient-specific factors, including the stability of the ongoing anticoagulant therapy, the bleeding risk profile (particularly gastrointestinal), and the severity of frailty, all within a framework of shared, multidisciplinary decision-making.

## 9. Knowledge Gaps and Future Directions

In the past, VKAs were the primary oral anticoagulant therapy for nonvalvular atrial fibrillation, while now DOACs are the standard of care. As a result of the progress of scientific research, novel therapeutic options are emerging for the future. In particular, factor XIa inhibitors may represent a promising alternative candidate for anticoagulation treatment, because, due to their selective mechanism of action, they have a reduced impact on hemostasis and may present a lower risk of bleeding than DOACs. If these benefits are validated, factor XIa inhibitors could represent an optimal strategy for patients at elevated risk of bleeding, such as frail older adults. Nevertheless, concerns persist regarding their potentially reduced efficacy in preventing thromboembolism compared to DOACs. In particular, among these molecules, Asundexian initially demonstrated a promising reduced risk of bleeding in comparison to apixaban in the phase 2 PACIFIC-AF study [126]. However, in the phase 3 OCEANIC-AF study, asundexian was found to be less effective in preventing stroke or systemic embolism compared to apixaban, leading to the premature termination of the trial [127]. Moreover, Abelecimab, a monoclonal antibody that inhibits factor XIa, offers the additional benefit of a long half-life, enabling once-monthly dosing and potentially improving adherence. In 1287 patients (average age 74 years old) enrolled in the phase 2 AZALEA-TIMI 71 study, abelecimab significantly reduced bleeding risk, particularly gastrointestinal bleeding, compared to rivaroxaban [128]. Although the difference was not statistically significant, ischemic stroke occurred more frequently in the abelecimab group. Additional phase 3 trials are needed to confirm whether abelecimab is non-inferior to DOACs in preventing stroke. Finally, a meta-analysis of all three RCTs mentioned above confirms that XIa inhibitors significantly reduce bleeding risk compared to DOACs in patients with AF, but their effectiveness in preventing thrombosis remains uncertain [129]. Further research is needed to support their implementation.

Finally, although this review mainly focuses on oral anticoagulation, percutaneous left atrial appendage occlusion (LAAO) may represent a valid alternative for older patients with AF who cannot take long-term anticoagulant [30]. However, frail patients are at higher risk for peri-procedural complications and adverse events [130,131]. Therefore, a multidisciplinary and individualized approach tailored to each patient’s risk profile is essential. Additionally, RCTs are required to definitively compare LAAO and DOAC in frail older adults.

## 10. Conclusions

In conclusion, frailty should not be considered a contraindication to anticoagulation. Instead, CGA is recommended to recognize and manage factors that may increase bleeding risk. The net clinical benefit generally favors anticoagulation, even though it diminishes with greater frailty severity. DOACs demonstrate a better safety profile compared to warfarin. Among DOACs, apixaban appears to be the safest option. Additionally, edoxaban at a very low dose may be effective for patients in whom standard anticoagulation is contraindicated. Furthermore, transitioning from VKAs to DOACs is a complex decision and should be based on the stability of the current anticoagulant treatment, bleeding risk, and frailty severity. Factor XIa inhibitors offer a lower bleeding risk than DOACs and are emerging as promising alternatives.

## Supplementary Materials

The following supporting information can be downloaded at https://www.mdpi.com/article/10.3390/jcm14228079/s1. Figure S1. Direct Oral Anticoagulants (DOACs) dosing and reduction criteria. Table S1. CHA_2_DS_2_-VA Score criteria for evaluation of Stroke Risk in AF patients.

## Abbreviations

The following abbreviations are used in this manuscript:

AF	Atrial Fibrillation
DOACs	Direct Oral Anticoagulants
VKAs	Vitamin K Antagonists
