# Supplementary material for: Anticoagulation in Frail Older Adults with Non-Valvular Atrial Fibrillation: Clinical Challenges and Personalized Approach
Source: J Clin Med. 2025 Nov 14;14(22):8079. doi: 10.3390/jcm14228079 (PMC12653972; doi:10.3390/jcm14228079)
Supplement: Supplementary file 1 [file jcm-14-08079-s001.zip › jcm-3967668-supplementary.pdf]

Figure S1. Direct Oral Anticoagulants (DOACs) dosing and reduction criteria

DOAC	Standard Daily Dose	Reduced Daily Dose	Criteria for Dose Reduction
**Apixaban**	5 mg twice daily (BID)	2.5 mg BID	Must meet at least two of the three criteria: age > 80 years, body weight < 60 kg, creatinine (Cr) > 1.5 mg/dL
**Dabigatran**	150 mg BID	110 mg BID	CrCl: 30–50 mL/min, OR Age >80 years
**Rivaroxaban**	20 mg once daily (QD)	15 mg QD	CrCl: 15–49 mL/min
**Edoxaban**	60 mg QD	30 mg QD	Must meet at least one of these criteria: CrCl: 15–50 mL/min, Body weight < 60 kg, OR concomitant use of potent P-glycoprotein inhibitors

Supplemental Table S1. CHA_2DS_2-VA Score criteria for evaluation of Stroke Risk in AF patients.

**Criteria**	**Score**
AGE	
65–74	+1
≥75	+2
Chronic Heart Failure	+1
Hypertension	+1
Prior stroke, TIA, or Thromboembolism	+2
Vascular disease	+1
Diabetes	+1
